# A Quantitative Assay for the Juvenile Hormones and Their Precursors Using Fluorescent Tags

**DOI:** 10.1371/journal.pone.0043784

**Published:** 2012-08-22

**Authors:** Crisalejandra Rivera-Perez, Marcela Nouzova, Fernando G. Noriega

**Affiliations:** Department of Biological Sciences, Florida International University, Miami, Florida, United States of America; University of Kentucky, United States of America

## Abstract

**Background:**

The juvenile hormones (JHs) are sesquiterpenoid compounds that play a central role in insect reproduction, development and behavior. The lipophilic nature of JHs and their precursors, in conjunction with their low concentration in tissues and susceptibility to degradation had made their quantification difficult. A variety of methods exist for JH quantification but few can quantify on the femtomole range. Currently applied methods are expensive and time consuming. In the present study we sought to develop a novel method for accurate detection and quantification of JHs and their precursors.

**Methods:**

A sensitive and robust method was developed to quantify the precursor, farnesoic acid (FA) and juvenile hormone III (JH III) in biological samples. The assay is based on the derivatization of analytes with fluorescent tags, with subsequent analysis by reverse phase high performance liquid chromatography coupled to a fluorescent detector (HPLC-FD). The carboxyl group of FA was derivatized with 4-Acetamido-7-mercapto-2,1,3-benzoxadiazole (AABD-SH). Tagging the epoxide group of JH III required a two-step reaction: the opening of the epoxide ring with sodium sulfide and derivatization with the fluorescent tag 4-(N,N-Dimethylaminosulfonyl)-7-(N-chloroformylmethyl-N-methylamino)-2,1,3-benzoxadiazole (DBD-COCl).

**Conclusions:**

The method developed in the present study showed high sensitivity, accuracy and reproducibility. Linear responses were obtained over the range of 10–20 to 1000 fmols. Recovery efficiencies were over 90% for JH III and 98% for FA with excellent reproducibility.

**Significance:**

The proposed method is applicable when sensitive detection and accurate quantification of limited amount of sample is needed. Examples include corpora allata, hemolymph and whole body of female adult *Aedes aegypti* and whole body *Drosophila melanogaster*. A variety of additional functional groups can be targeted to add fluorescent tags to the remaining JH III precursors.

## Introduction

The juvenile hormones (JHs) are sesquiterpenoid compounds that play a central role in insect reproduction, development and behavior [1]. They are synthesized and secreted by the *corpora allata* (CA), a pair of endocrine glands with neural connections to the brain [2]. The biosynthesis of JH is divided into early and late steps [3]. The early steps follow the mevalonate pathway from acetyl-CoA to farnesyl pyrophosphate (FPP). The late steps involve the hydrolysis of FPP to farnesol [4], followed by oxidation to farnesal [5] and farnesoic acid (FA) [6]. FA is finally converted to JH III by means of a methyl transfer [7] and epoxidation [8].

The lipophilic nature of JHs, in conjunction with their low concentration in tissues, susceptibility to degradation and their tendency to bind non-specifically has made difficult their quantification. Three methods have been traditionally employed to quantify JHs from biological samples: 1) bioassays, 2) radioimmunoassay (RIA) and 3) physicochemical assays [1]. In addition, a radiochemical assay (RCA) has been used extensively to measure JH synthesis in the isolated CA [9].

**Figure 1:**
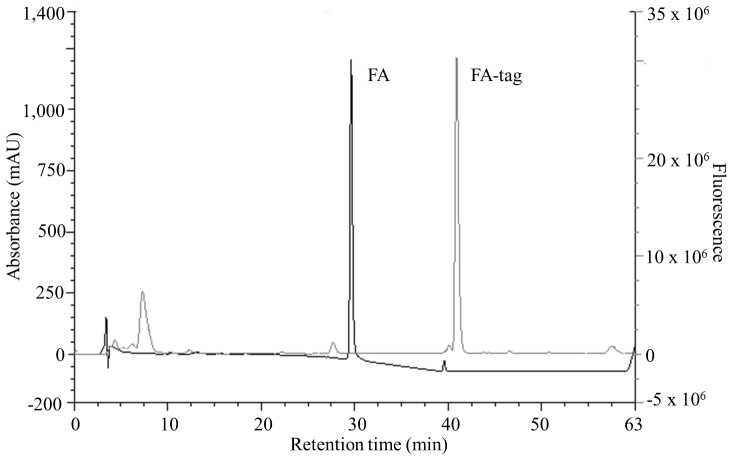
FA HPLC-FD chromatograms. FA (100 pmol) samples before and after derivatization are superimposed. FA (black line) and FA derivatized with AABD-SH (FA-tag, grey line). Left Y axes: UV absorbance, right Y axes: fluorescence.

**Figure 2:**
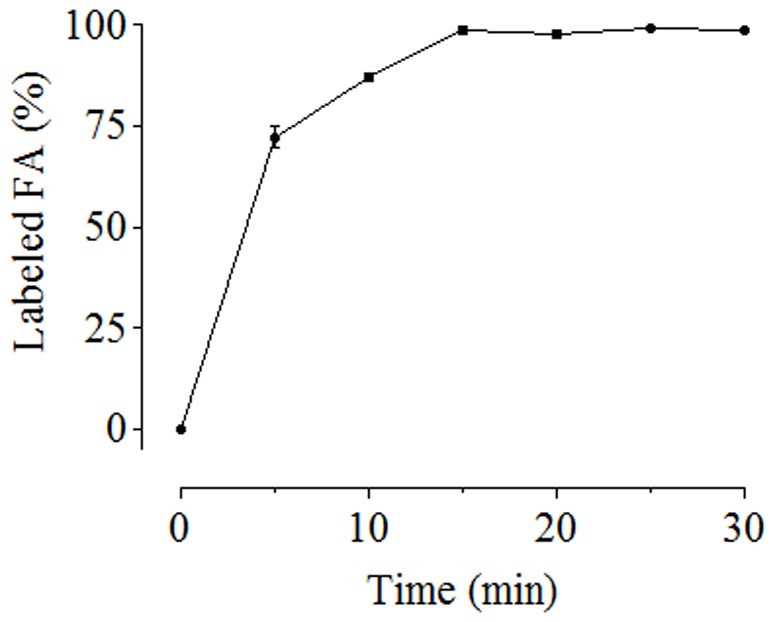
The efficiency of FA derivatization with AABD-SH. Fluorescence signal increases with time of incubation up to 15 minutes. Each point represents the percentage of tagged FA peak area measured by HPLC-FD. Data represent the means ± SD of three independent experiments.

The first measurements of JHs were done using bioassays. Biological extracts were injected into insects (mostly Lepidopteran pupae) and hormonally induced phenotypes, such as disruption of metamorphosis, were evaluated [10], [11]. These assays were valuable, but laborious and lacked specificity. Radioimmunoassays (RIA) were developed in the 1980s as an alternative methodology for JH quantification [12], [13], but their high variability and the cross-reactivity of antibodies against the various JHs has been criticized [2], [13]. Physicochemical methods include gas chromatography coupled with mass spectrometry (GC-MS) [14], [15], liquid chromatography tandem mass spectrometry (LC-MS/MS) [16]-[18], ion-trap MS operated in chemical ionization mode [19] nuclear magnetic resonance [20], infrared spectroscopy [21] or rapid direct analysis in real time mass spectrometry (DART-MS) [22]. Analytical methods coupled to mass spectrometry provide unequivocal identification and quantification of the compounds and are therefore considered the most accurate for the analysis of JH [13], [23]. However, MS approaches are expensive, complicated and often have detection limits only in the picogram or nanogram range [14], [24], [25]. The radiochemical assay (RCA) is a sensitive technique for the precise determination of JH synthesis rates. It measures the incorporation of the methyl group from [^3^H]methyl methionine into JH in isolated CA [9], [26], [27]. The use of RCA is limited to *in vitro* assays and problems such as contamination of radiolabeled methionine and lack of accuracy have been reported [28]. JHs and their precursors differ markedly in structure and physical properties and finding simple alternative protocols for quantification has been challenging [25], [29]–[31].

High performance liquid chromatography coupled to fluorescent detection (HPLC-FD) is a well-established sensitive method for the accurate detection of low concentration of metabolites [32]. Most analytes lack natural fluorescence, therefore derivatization with fluorescent tags enhances the detectability of these compounds to the low fmol range [33]. Various fluorescent labeling reagents have been developed for tagging functional groups such as carboxyl, hydroxyl and thiol [34], [35]. In this study a sensitive and robust method was developed to quantify FA and JH III in biological samples. This assay combined the advantages of fluorescent tag detection of the derivatized analytes with the use of an HPLC coupled to a fluorescent detector to allow quantitative analysis of the analytes. Extracted analytes are directly labeled with fluorogenic labeling reagents in sealed reaction vials. Linear responses were obtained over the range of 10–20 to 1000 fmols. FA and JH III levels were quantified from corpora allata, hemolymph and whole body of female adult *Aedes aegypti*. JH III and JH III bisepoxide (JHB_3_) were detected in whole body extracts of adult *Drosophila melanogaster*.

**Figure 3:**
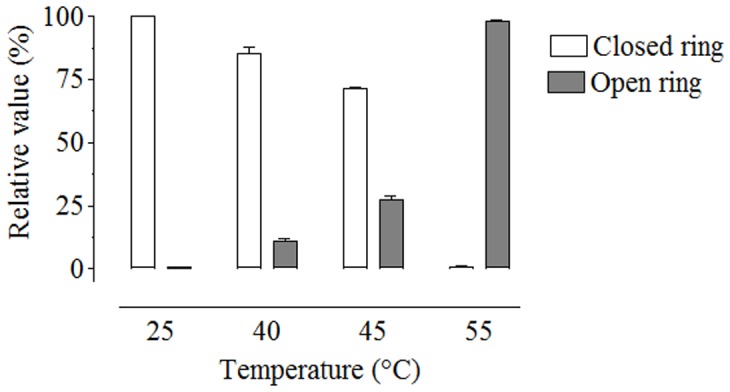
Effect of temperature on the opening of the epoxide ring of JH III. JH III was incubated with sodium sulphide (100 mM) at different temperatures. White bars indicate the percentage of intact JH III after the reaction and dark bars indicate JH III with open rings after the reaction. Data represent the means ± SD of three independent experiments.

## Materials and Methods

### 2.1 Insects

*Aedes aegypti* of the Rockefeller strain were reared at 28°C and 80% relative humidity under a photoperiod of 16 h light: 8 h dark. Mated adults were offered a cotton pad soaked in 3% sucrose solution. The cotton pad sucrose-fed adults are referred to as sugar fed. *Drosophila melanogaster* w^118^ stocks were reared at 22°C on standard agar molasses medium.

**Figure 4:**
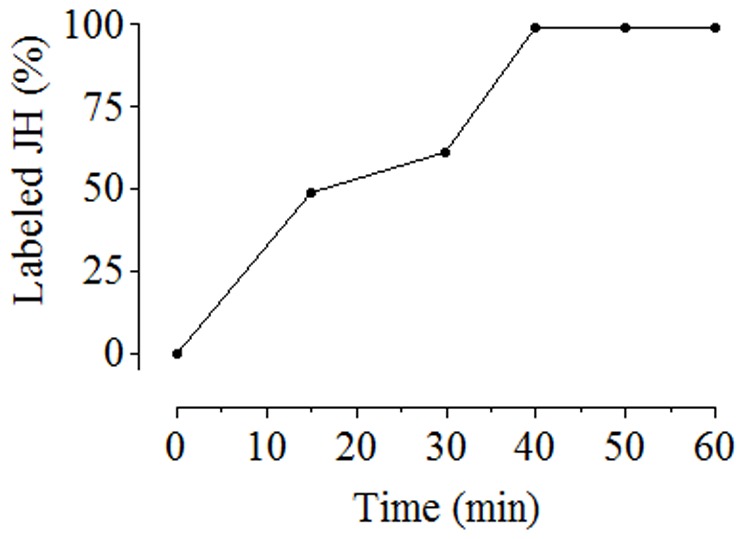
The efficiency of derivatization of the JH III epoxide ring with DBD-COCl. Fluorescence signal increases with time of incubation up to 40 minutes. Each point represents the percentage of tagged JH III peak area measured by HPLC-FD. Data represent the means ± SD of three independent experiments.

**Figure 5:**
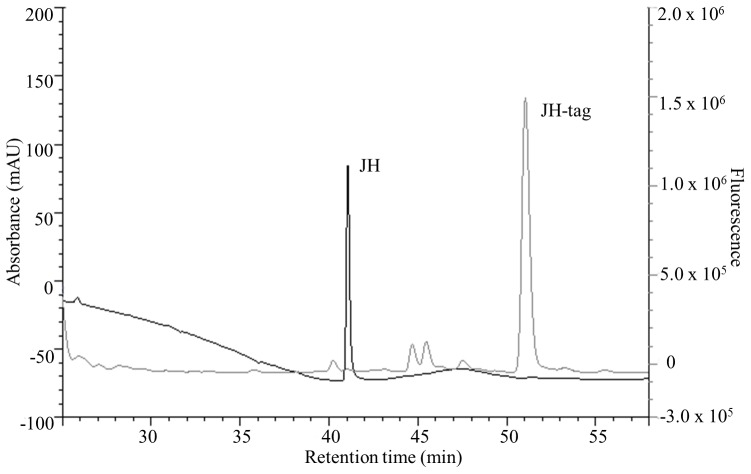
JH III HPLC-FD chromatograms. JH III (100 pmol) samples before and after derivatization are superimposed. JH III (black line) and JH III derivatized with DBD-COCl (JH-tag, grey line). Left Y axes: UV absorbance, right Y axes: fluorescence.

### 2.2 Reagents and chemicals

HPLC-grade methanol, acetonitrile, juvenile hormone III, triphenylphosphine (TPP), 2,2′–dipyridyl disulfide (DPDS), citronellol and dichloromethane were obtained from Sigma-Aldrich (St. Louis, MO). Farnesoic acid (Echelon, Salt Lake City, UT), sodium sulfide nonahydrate (MP Biomedicals, Solon, OH), DBD-COCl (4-(N,N-Dimethylaminosulfonyl)-7-(N-chloroformylmethyl-N-methylamino)-2,1,3-benzoxadiazole) and AABD–SH (4-Acetamido-7-mercapto-2,1,3-benzoxadiazole) were from TCI-America (Portland, OR). JHB_3_ was a gift from Dr. Stephen Tobe and was synthesized from methyl farnesoate using m-chloroperbenzoic acid in dichloromethane [36]. Diagnostic ions used for identification of JHB III included m/z  = 300, 283, 265, 251 and 301 as reported by Yin et al. [37].

**Figure 6:**
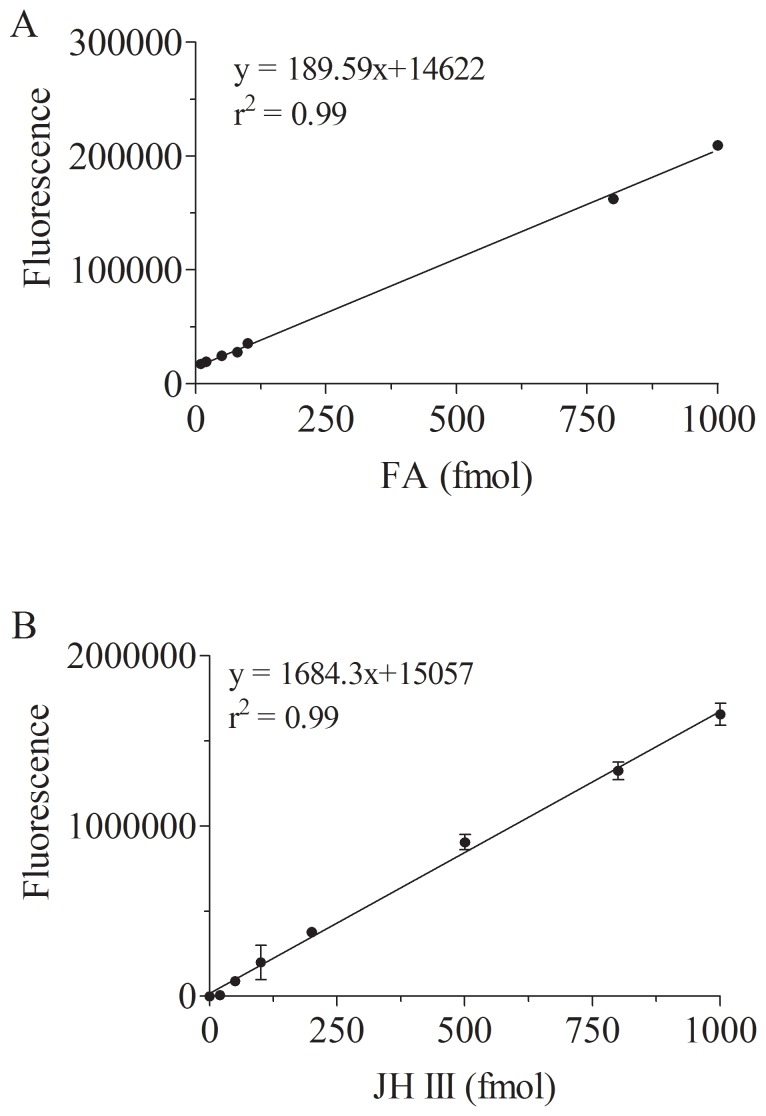
Relationship between FA and JH III concentrations after derivatization and fluorescence intensities. A) FA. B) JH III. Data represent the means ± SD of three independent experiments.

**Table 1:** Intra-run, inter-run and day run precision of JH III and FA quantification.

Compound	Intra-run^a^ (% RSD)	Inter-run^b^ (% RSD)	Day run^c^ (% RSD)
JH III	3.4	3.74	5.46
FA	1.53	5.57	3.89

a Three and six measurements of one sample respectively, ^b^ three separately extracted samples, two determination each, ^c^ three measurements one day after the reaction from three separately extracted samples, two determination each. RSD: relative standard deviation.

### 2.3 Stock solutions

Stock solutions were prepared as follow: AABD-SH (10 mM) in dichloromethane, TPP (5 mM) and DPDS (5 mM) in acetonitrile and DBD-COCl (1 mM) in chloroform. Solutions were protected from light with aluminum foil and stored at 4°C until used. Under these conditions, solutions were stable for at least one month. Sodium sulfide was dissolved with water to a final concentration of 100 mM. Sodium sulfide solutions were stable for 3 days. Stock solutions of JH III and FA were prepared in methanol and stored at −20°C.

**Table 2:** Recovery of labeled FA and JH.

Compound	Input amount (fmol)	Mean amount (fmol/CA)	% Recovery
FA	0	77.30±2.42	-
	50	128.00±0.56	100.0
	100	176.10±6.90	99.37
	1000	1058.35±7.63	98.20
JH III	0	45.25±2.42	-
	50	91.25±0.56	92.4
	100	137.3±8.90	94.3
	1000	1025±7.63	98.06

24h CA-CC (n = 10 for FA and n = 3 for JH). Measurements were done by duplicate.

### 2.4 Fluorescent tagging of FA, JH III and JHB_3_

#### 2.4.1 FA tagging

In a 2 ml glass tube 20 µl of FA was mixed with 20 µl of 10 mM AABD-SH, 20 µl of 5 mM TPP and 20 µl of 5 mM DPDS. Vials were allowed to stand for 15 min at room temperature and 20 µl of acetonitrile was added to a final volume of 100 µl (Figure S1). Aliquots of the reaction mixtures were analyzed by HPLC-FD.

**Figure 7:**
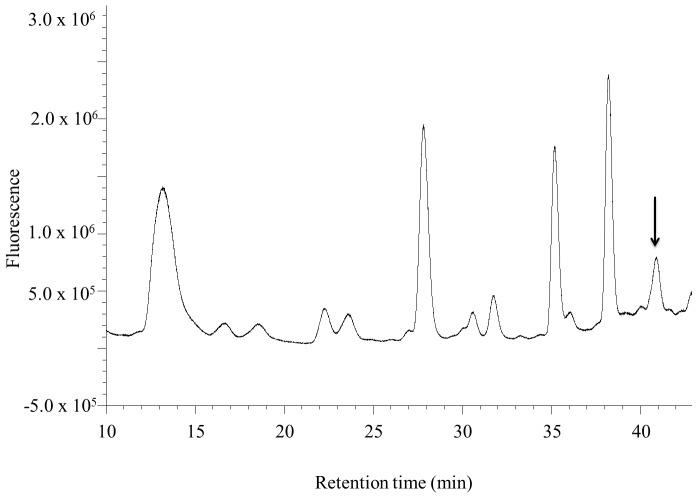
Detection of tagged FA in mosquito CA-CC by HPLC-FD. Extracts of CA-CC from 24 h sugar-fed females derivatized with AABD-SH. The fluorescent peak with a retention time of 40.9 min represents FA (arrow).

#### 2.4.2 JH III tagging

Tagging of JH III required a two-step reaction. A) *Opening of the epoxide ring*: In a 2 ml glass tube 10 µl of JH III were mixed with 100 µl of 100 mM sodium sulfide. Tubes were heated in a water bath at 55°C for 30 min and then cooled to room temperature. B) **Derivatizing with a fluorescent tag.** After the epoxide ring was opened, 50 µl of 1 mM DBD-COCl in chloroform were added and samples were incubated for 40 min at room temperature, protected from light and slightly agitated. Reactions were quenched with 90 µl of acetonitrile to a final volume of 250 µl. (Figure S1). Aliquots of the reaction mixtures were analyzed by HPLC-FD.

**Figure 8:**
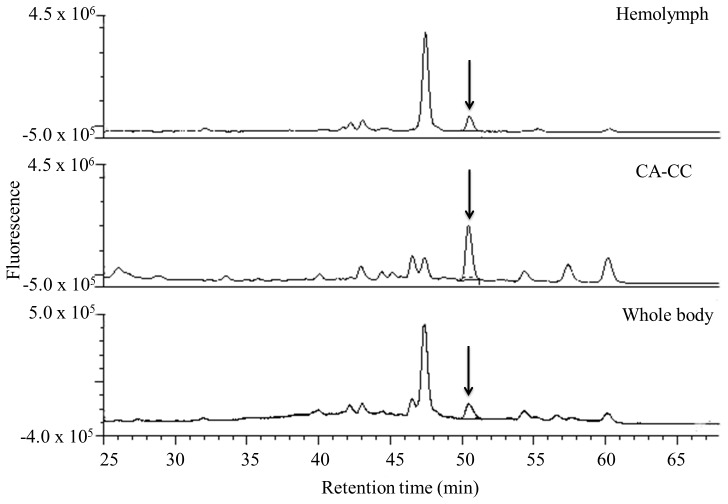
Detection of tagged JH III in mosquito samples by HPLC-FD. Extracts of hemolymph (diluted 1∶10), CA-CC and whole body (diluted 1∶20) from 24 h sugar-fed females were derivatized with DBD-COCl. The fluorescent peak with a retention time of 51 min represents JH III (arrows).

**Figure 9:**
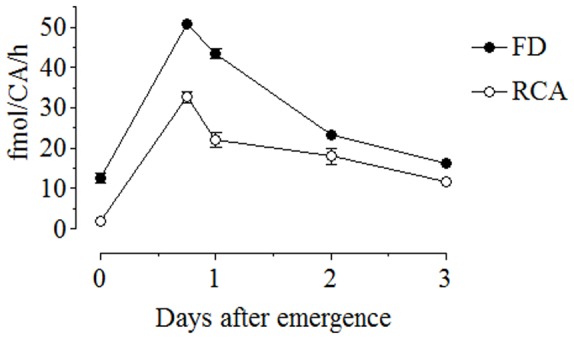
Biosynthesis of JH III *in vitro* in sugar-fed females. CA-CC complexes from sugar-fed females were dissected at different times after emergence, incubated for 4 h, extracted and analyzed by HPLC-FD. Radio chemical analysis (RCA) data are from Li et al. [31]. Data represent the means ± SD of three independent experiments. HPLC-FD: (**•**), RCA: (○).

#### 2.4.3 JHB_3_ tagging

For tagging JHB_3_ we used a similar protocol to that described for JH III, with the following modifications, 1 h incubation in 100 mM sodium sulfide at 55°C for opening of the epoxide rings and 1h incubation with 1 mM DBD-COCl in chloroform at room temperature for derivatization.

Characterization of standards and tagged compounds were performed by electrospray ionization-liquid chromatography-mass spectrometry (ESI-LC/MS) using a LCQ Deca XP Max (Finnigan) ion trap mass spectrometer. Optimal conditions were set as follows: capillary temperature 275°C with a 35 l/min flow rate and ionization voltage of 5 kV. All spectra were obtained in the positive ion mode over a mass range of m/z 150–1500.

### 2.5 In vitro labeling FA, JH III and JHB_3_ from biological samples

#### 2.5.1 Analysis of corpora allata-corpora cardiaca (CA-CC) samples

CA-CC of female adult *A. aegypti* were isolated as described by Li et al. [31]. For FA quantifications, 10 pairs of CA-CC complexes were dissected into 150 µl of saline solution (2 ml vials) and 500 µl of hexane were added. The vials were vortexed for 1 min, sonicated for 5 min, vortexed again for 1 min and centrifuged at 2000 *g* for 5 min at 4°C. The organic phases (upper layer) were removed and transferred to new vials. The extracted organic phases (∼500 µl) were filtered with Nalgene 4-mm syringe filters (0.2 µm nylon membrane, #176) and dried under a N_2_ atmosphere. Samples were stored at −20°C until used. The samples were tagged as described in section 2.4.1 and aliquots of the labeled reactions were analyzed by HPLC-FD.

For JH III quantification, 10 pairs of CA-CC complexes were dissected and incubated for 4 h in the dark at 32°C under continuous gentle agitation in tissue culture medium M-199 (Lavallette, NJ, USA) containing 2% Ficoll, 25 mM HEPES (pH 6.5) and methionine (50 µM). After incubation 150 µl of 100 mM sodium sulfide was added and the epoxide ring was opened by heating the biological extracts for 30 min at 55°C. Afterwards, samples were extracted using hexane as described for FA quantification. The recovered organic phase (∼500 µl) was filtered with a Nalgene filter (0.2 µm nylon membrane, #176) and dried under N_2_ atmosphere. Samples were stored at −20°C until derivatization. For fluorescent tagging, samples were reconstituted with 25 µl of acetonitrile and 25 µl of 1 mM DBD-COCl were added. Labeling mixtures were incubated at room temperature for 40 min and reactions were terminated by adding 50 µl of acetonitrile. Aliquot of the reactions were analyzed by HPLC-FD.

#### 2.5.2 Analysis of insect whole body extracts

For JH III quantification, 10 adult female mosquitos (24 h after emergence) were processed by the method described by Bergot et al. [14] that includes an acetonitrile/pentane extraction and a C18 solid-phase extraction cartridge purification (Figure S2). The recovered organic fraction was reduced to a volume of a 100 µl and the JH III epoxide ring was opened by the addition of 150 µl of sodium sulfide and incubation at 55°C for 30 min. Samples were then extracted with hexane; the recovered organic phase (∼500 µl) was filtered with a Nalgene filter (0.2 µm nylon membrane, #176), dried under N_2_ and stored at −20°C until used. The labeling of JH III with fluorescent a tag was done as described for CA-CC (Figure S2).

#### 2.5.3 Analysis of hemolymph samples

Hemolymph was collected from 10 sugar-fed female mosquitos (24 h after emergence) by perfusion with *Aedes* physiological saline as described by Qayum and Telang [38] Hemolymph samples were processed as described in section 2.5.1. The labeling of JH III with a fluorescent tag was done as described for CA-CC.

### 2.6 HPLC-FD analysis

HPLC-FD was performed using a Dionex Summit System (Dionex, Sunnyvale, CA) equipped with a 680 HPLC pump, a TCC 100 column oven, a UV 170U detector and an UltiMate 3000 fluorescence detector connected in series and a Chromeleon software version 6.8 SR10. The separation of tagged compounds was performed on an analytical column Acclaim 120 C18 (250×2.1 mm ID, particle size 5 µm) (Dionex), using isocratic elution from 0 to 20 min (acetonitrile/water, 1 to 1 v/v), followed by a linear gradient from 20 to 50 min (acetonitrile-water (50 to 95%, v/v) and another isocratic elution from 50 min (acetonitrile, 95%). Flow rate was 0.2 ml/min and column temperature was 25°C. The eluate was monitored with UV (214 nm) and fluorescence detection with the following wavelengths for excitation and emission: FA (λ_excitation._ 368 nm; λ _emission._ 524 nm) and JH III (λ _ex._ 450 nm; λ _em._ 560 nm).

### 2.7 Recovery efficiency for FA and JH III

The efficiency of sample recovery was investigated using two independent strategies:

1. The addition of 100 ng of citronellol as an internal standard to the samples before derivatization with subsequent analysis of citronellol recovery using the UV detector (214 nm).
2. The spiking of a biological sample (CA-CC) before extraction with known amounts of the analyte (0, 50, 100 and 1000 fmols of FA or JH III). The recovery was expressed as a percentage and was calculated by subtracting the endogenous amount of analyte from the amount found, divided by the amount spiked and multiplying by 100.

### 2.8 Reproducibility of the HPLC-FD method

The linear relationship between analyte concentration and the area of the HPLC-FD signal was verified by three replicate analyses of three calibration standard curves (10–1000 fmols). *Intra-run* analysis assesses the reproducibility between independent HPLC runs of the same sample; this was done by 3–6 independent measurements of FA and JH III of the same biological sample. *Inter-run* variability was evaluated by extracting, tagging and analyzing three different biological samples by duplicate. *Day-run* reproducibility assesses the effect of analyzing samples for two consecutive days. This was done by running three independent biological samples on two consecutive days. Reproducibility was calculated as the Relative Standard Deviation (RSD), obtained by dividing the standard deviation by the average and then multiplied by 100 to be expressed as a percentage.

## Results

### 3.1 Optimal conditions for the addition of fluorescent tags to FA and JH III

#### 3.1.1 Tagging FA

FA was derivatized with AABD-SH at room temperature in the presence of TPP and DPDS (Figure S2). The process resulted in the formation of a higher-molecular-weight fluorescent derivative. Retention time increased from 29.6 min to 39.6 min for FA with UV detection (214 nm) and resulted in a retention time of 40.9 min with fluorescence detection (Fig. 1). The delay of 1.2 min of the precursor FA tagged between UV and FD signals is because the FD detector is connected in series after the UV detector.

The optimal time for the derivatization reaction of FA was determined to be 15 min. The amount of labeled FA reached a plateau after 15 min, indicating that in the described conditions the thiol group reacted rapidly with the carboxyl group of FA (Fig. 2).

#### 3.1.2 Tagging JH III

Tagging the epoxide group of JH III required a two-step reaction: the opening of the epoxide ring and derivatization with the fluorescent tag (Figure S2). Optimal conditions for the opening of the epoxide ring were determined by analyzing changes in temperature. Incubation in the presence of 100 mM sodium sulfide at 55°C for 30 min resulted in over 98% of ring opening (Fig. 3). In addition, the JH III epoxide ring could also be opened using either sodium sulfide titrated at pH 10 for 30 min at room temperature, 0.02 M HCl for 4 h at room temperature or 0.05 N H_2_SO_4_ overnight at 40°C.

After the JH epoxide ring was opened, derivatization with the fluorescent tag was done at room temperature with DBD-COCl. The optimal time of labeling was 40 min (Fig. 4). The opening of the epoxide ring increased JH III retention time from 26.5 min to 41.0 min (UV detector, 214 nm); the tagged JH III had a retention time of 51 min on the fluorescence detector (Fig. 5).

The identities of JH III and tagged JH III were verified by LC-MS. JH III was characterized based on the diagnostic ions (m/z 267, 249, 235, 217 and 189) as described by Chen et al. [25]. The molecular ion of JH III after ring opening had a m/z 284 and after labeling with DBD-COCl had a m/z  = 928.9, suggesting that the ring of JH III is transformed to the diol form and each hydroxyl group is tagged with a molecule of DBD-COCl (theoretical mass: 928.7).

#### 3.1.3 Tagging JHB_3_

Since JHB_3_ has two epoxide rings that are targets for derivatization, we extended the reaction times for ring opening and tagging to 1 h each. After derivatization two main peaks with retention times of 50 and 60.3 min were observed (Figure S3). The identity of the tagged JHB3 was verified by LC-MS. The tagged JHB3 had an m/z  = 944.4, suggesting that two tags are linked to the molecule (theoretical mass: 943.4).

### 3.2 Reproducibility and limit of detection of the HPLC-FD method

Calibration curves ranging from 10 to 1000 fmol had linear relationships between fluorescent signal integrated peak areas and FA or JH III concentrations (R^2^ of 0.99) (Fig. 6). Limit of quantifications were 10 and 20 fmol for FA and JH III respectively, with a signal to noise ratio of 7.

The reproducibility of the technique was evaluated by measuring the amount of FA present in extracts of CA-CC dissected from adult female mosquitoes 24 h after emergence and by measuring the amount of JH III secreted into the medium when similar CA-CC were incubated *in vitro* for 4 h. Reproducibility was evaluated by calculating the RSD. Intra-run, inter-run and day-run changes were low, with RSDs below 6% for FA and JH III (Table 1).

### 3.3 Recovery efficiencies for FA and JH III

FA recovery was 95% based on citronellol addition and 98% when evaluated by measuring the amount of FA present in extracts of CA-CC dissected from adult female mosquitoes 24 h after emergence and spiked with increasing amounts of FA (0, 50, 100 and 1000 fmol) (Table 2). JH III recovery efficiency was over 90% when CA-CC were incubated *in vitro* for 4 h and then spiked with increasing amounts of JH III (0, 50, 100 and 1000 fmol) (Table 2).

### 3.4 Measurement of FA and JH III in biological samples

FA was quantified in CA-CC complexes dissected from adult female mosquitoes 24 h after emergence, tagged with AABD-SH for 15 min at RT and analyzed by HPLC-FD. FA was detected by the fluorescence detector with a retention time of 41.0 min (Fig. 7). The amount of FA in CA-CC extracts was 77.3±4.1 fmol/CA. JH III levels were below detection range in CA-CC extracts because JH III is secreted immediately after synthesis [31].

Tagged JH III was quantified in three types of female adult mosquito samples: hemolymph, whole body extracts and incubated CA-CC + medium (Fig. 8). JH III levels in hemolymph and whole body are expressed relative to mosquito wet weight and were 1.4±0.04 pg/g for hemolymph and 801±0.3 pg/g for whole body extracts. In addition we analyzed the rates of JH III biosynthesis of CA-CC dissected from sugar-fed adult female mosquitoes at different times after adult emergence. These values were very similar to those previously reported using the RCA [31] (Fig. 9).

We also quantified JH III levels in whole body extracts of *D. melanogaster* adult females (1.17±0.06 pmol/g) (n = 2) and males (0.92±0.17 pmol/g) (n = 2). These values were the results of pooled samples of adults of different ages (56 females and 26 males respectively) and were similar to those previously reported [39].

## Discussion

Fluorescence has emerged as a valuable tool in metabolomic studies since it is possible to detect and quantify trace-level compounds by their intrinsic fluorescence or after labeling them with an extrinsic fluorophore [40]. Most compounds do not possess natural fluorescence so derivatization with fluorescent labeling reagents can be utilized to enhance their detectability. Typically, fluorescent labeling reagents are composed of a highly fluorescent group and a reactive group that reacts with the functional group of the target compound. The detection limit of the derivatized analyte is determined by the fluorophore brightness allowing the detection in the fmol range.

### 4.1 Analysis of the precursors of JH biosynthesis

Our goal was to develop a sensitive, simple and robust technique to measure JH III biosynthetic precursors in the femtomole range. In the CA of mosquitoes there are 14 recognized precursors of JH III. They are structurally diverse and have different functional groups suitable for derivatization with fluorescent tags. Carboxylic groups are one of the most common functional groups in nature and excellent targets for tagging. There are five precursors with carboxyl groups: hydroxymethylglutaryl-CoA, mevalonate, mevalonate phosphate, mevalonate diphosphate and FA. Farnesoic acid was chosen as a proof of principle for the optimization of the technique. The HPLC-FD protocol has high sensitivity (10 fmol), outstanding recovery and excellent reproducibility allowing for the quantification of the FA pool in a single CA. In labeling FA the correct sequence of addition of the condensation reagents (TPP and DPDS) is important; DPDS should be added last to the reaction, otherwise tagging is not properly completed. A similar observation was described by Uchiyama [41]. We also successfully tagged the carboxyl groups of 4 additional precursors and detected hydroxymethylglutaryl-CoA, mevalonate, mevalonate phosphate and mevalonate diphosphate in mosquito CA-CC extracts (Figure S4). Additional unknown compounds are labeled, but identification of the target compounds is conclusive based on retention times.

A variety of additional functional groups can be targeted to add fluorescent tags to the other JH III precursors. We labeled and detected the thiol group of acetyl-CoA and acetoacetyl-CoA, the hydroxyl group of farnesol and the aldehyde group of farnesal (Figure S4). The phosphate group of isopentenyl-PP, dimethylallyl-PP, geranyl-PP and farnesyl-PP and the ester group of methyl farnesoate could also be targeted for derivatization.

### 4.2 Analysis of JH levels

Choosing the right extraction method was critical for the success of the technique. Several solvents have been described for the extraction of JH: methanol-water-hexane [12], [42], chloroform [43] and isooctane [44]. When extracting JH III from CA-CC, hexane was almost two-fold more effective than chloroform. For the analysis of whole body insect extracts the protocol described by Bergot et al [14] was 20-fold more efficient than methanol/hexane (data not shown). Quantification by derivatizing epoxide groups with fluorescent tags was previously reported by Sano and Takitani [45]; their method was based on opening the epoxide ring with hydrogen sulfide in the presence of sodium [46] and converting the epoxide into a fluorescent isoindole adduct by treating the derivative with *o*-phthalaldehyde (OPA) and taurine [45]. Duchateau et al. [47] reported a HPLC method with a detection limit of 2 pmol based in the same principle. Unfortunately OPA derivatives are unstable and fluorescent products degrade after 15 min [48]. We modified the tagging step of the protocol in order to improve the stability, increase the sensitivity to the femtomole range and detect JH III in biological extracts. We are reporting for the first time the tagging of an epoxide ring using DBD-COCl. We selected DBD-COCl instead of OPA, since DBD-COCl can react with nucleophile groups under mild conditions. The tagging produced a stable JH III derivative that was readily detected by HPLC-FD. Epoxides are known to give the corresponding thioglycol derivative when treated with hydrogen sulfide [46]; the reaction involves a nucleophilic attack at the sterically less hindered site of the epoxide generating a sulfhydryl group at C11 and a hydroxyl group at C10 suitable for tagging with DBD-COCl [49]. This protocol is very effective, however a hydroxyl group suitable for tagging by production of a diol form of JH III could also be achieved using HClO_4_ in tetrahydrofuran [50] or H_2_SO_4_ [12].

Opening of the epoxide ring converts JH III into a diol form. The diol is more hydrophilic and less adsorbed onto surfaces (less “sticky”); it is also easier to separate from other lipids that are very abundant in insect samples [12]. By opening the epoxide ring at the beginning of the sample preparation, JH loses were significantly reduced thereby achieving excellent recovery efficiencies. We believe this striking increase in the stability and recovery of JH is one of the strengths of this protocol. Once the epoxide ring has been opened samples can be stored or shipped without significant losses.

Reported levels of JH in hemolymph or insect whole body extracts vary between 20 and 4500 pg/ul [29], [51] and levels of JH biosynthesis by isolated CA are between 110 fmol and 50 pmol per CA/h [52], [53]. Our protocol works well in this range and was validated by confirming JH levels previously reported using other methods. The changes in levels of JH III synthesis by the isolated CA-CC of adult female mosquito were similar to those previously described using RCA by Li et al. [31], although HPLC-FD had consistently higher values and less variability than RCA [31]. Variability when doing RCA is mostly the result of losses during extractions, thin layer chromatography separations and scintillation cocktail quantification. The levels in whole body extracts were similar to those reported using GC-MS for *A. aegypti* [29] and *D. melanogaster* [39], emphasizing the usefulness of HPLC-FD for analyzing JH levels in insect samples. The HPLC-FD protocol is simple, fast and relatively inexpensive. When quantifying JH from hemolymph or CA samples, the total time needed to extract the sample, open the ring and derivatize the sesquiterpene was less than 90 min and multiple samples can be processed simultaneously. Quantification from whole body samples is more laborious and takes around 6 h, but extracting JH from whole body extracts is cumbersome regardless of the analytical method used afterwards to quantify sesquiterpenes.

### 4.3 Conclusions

HPLC-FD offers several important advantages that include sensitivity, specificity and reproducibility. By targeting different functional groups all JH precursors can be labeled and quantified. Most of them can be labeled with benzofurazan derivatives allowing the simultaneous detection in a single HPLC run. The HPLC-FD protocol described could be further optimized. New chromatographic techniques, based on sub-micron particle sizes, such as ultra-high performance liquid chromatography (UHPLC), would allow rapid separations (5 min or less) with higher peak capacities reducing the time of analysis and increasing resolution. This protocol could also be adapted for the high-throughput analysis of samples using multiwell plates and fluorescent spectrophotometers as long as most of the fluorescent signal corresponds to the targeted labeled analyte. In summary, this technique promises to become a useful tool to the comprehensive analysis of intracellular metabolites in insects.

## Supporting Information

Figure S1 **Fluorescent tagging of JH III and FA.** *Tagging JH III*: Tagging of JH III required a two-step reaction. First step: Opening of the epoxide ring (blue) with sodium sulfide at 55°C to form a JH diol. Second step: Derivatizing with DBD-COCl to form a higher-molecular-weight fluorescent derivative (the fluorescent tag is shown in blue). *Tagging FA*: The carboxylic group of FA (blue) was derivatized with AABD-SH at room temperature in the presence of triphenylphosphine (TPP) and 2,2′-dipyridyl disulfide (DPDS). The process resulted in the formation of a higher-molecular-weight fluorescent derivative (the fluorescent tag is shown in blue).
(PDF)

Figure S2 **In vitro labeling JH III from biological samples.** I) Extraction protocol: Insect tissues were processed by the method described by Bergot et al. (1981) that includes an acetonitrile/pentane extraction and a C18 solid-phase extraction cartridge purification. The recovered organic fraction was reduced to a volume of a 100 µl and the JH III epoxide ring was opened by the addition of 150 µl of sodium sulfide and incubation at 55°C for 30 min. Samples were then extracted with hexane; the recovered organic phase (∼500 µl) was filtered with a Nalgene filter (0.2 µm nylon membrane), dried under N2 and stored at −20°C until used. II) Opening epoxide ring: The epoxide ring was opened by the method described by Duchateau and Jacquemin (1993). After extraction, 150 µl of 100 mM sodium sulfide was added and the epoxide ring was opened by heating the biological extracts for 30 min at 55°C. Afterwards, samples were extracted using hexane. The recovered organic phase (∼500 µl) was filtered with a Nalgene filter (0.2 µm nylon membrane) and dried under N_2_ atmosphere. III) Labeling with a fluorescent tag: For fluorescent tagging, samples were reconstituted with 25 µl of acetonitrile and 25 µl of 1 mM DBD-COCl were added. Labeling mixtures were incubated at room temperature for 40 min and reactions were terminated by adding 50 µl of acetonitrile. Aliquot of the reactions were analyzed by HPLC-FD.
(TIF)

Figure S3 **Tagging and detection of JHB_3._** JHB3 was a gift from Dr. Stephen Tobe and was synthesized from methyl farnesoate using m-chloroperbenzoic acid in dichloromethane (Bendena et al., 2011). Isolated JHB3: JHB3 was derivatized with AABD-SH. Two JHB3 fluorescent peaks with retention times of 50 and 60.3 were detected (arrows). Drosophila (whole body): JH III and JHB3 detection in whole body extracts of a pool of 56 D. Melanogaster adult females of different ages/JH III is marked with a large arrow. The two JHB3 peaks are marked with small arrows.
(TIF)

Figure S4 **JH pathway precursors derivatized with fluorescent tags.** A variety of additional functional groups can be targeted to add fluorescent tags to the other JH III precursors. We labeled and detected the thiol group of acetyl-CoA and acetoacetyl-CoA with DBD-H ( = 4-(N,N-Dimethylaminosulfonyl)-7-hydrazino-2,1,3-benzoxadiazole) at Exc/Em: 450/565 nm, the hydroxyl group of farnesol with DBD-COCl (4-(*N*,*N*-Dimethylaminosulfonyl)-7-(*N*-chloroformylmethyl-*N*-methylamino)benzofurazan) at Exc/Em: 443/546, the carboxyl group of HMG-CoA, mevalonate, phosphomevalonate and diphosphomevalonate with AABD-SH ( = 4-acetamido-7-mercapto-2,1,3-benzoxadiazole) at Exc/Em: 368/524 nm, and the aldehyde group of farnesal with NBD-H ( = 4-hydrazino-7-nitro-2,1,3-benzoxadiazole hydrazine) at Exc/Em: 450/565. The precursor were eluted by reverse phase-HPLC coupled with a fluorometer detector at the same conditions described for JH and farnesoic acid.
(TIF)
