# Supplementary material for: A Quantitative Assay for the Juvenile Hormones and Their Precursors Using Fluorescent Tags
Source: PLoS One. 2012 Aug 22;7(8):e43784. doi: 10.1371/journal.pone.0043784 (PMC3425502; doi:10.1371/journal.pone.0043784)

Juvenile hormone III

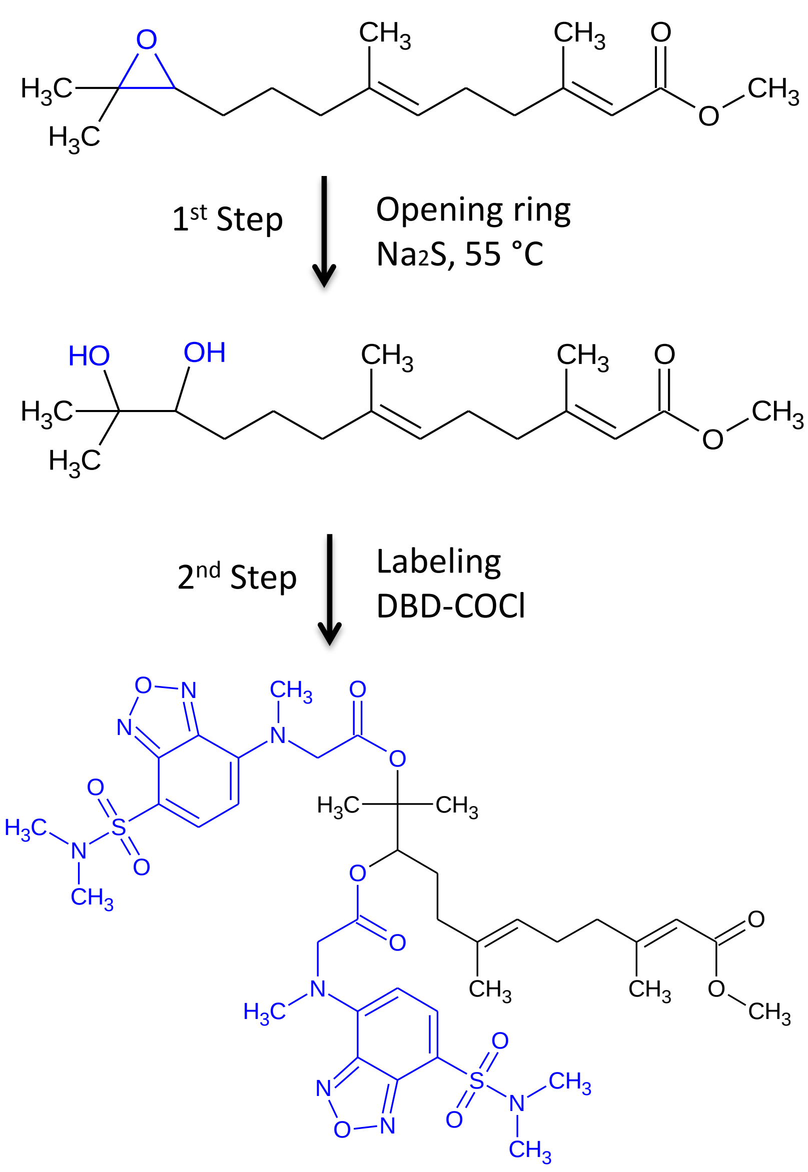

Farnesoic acid

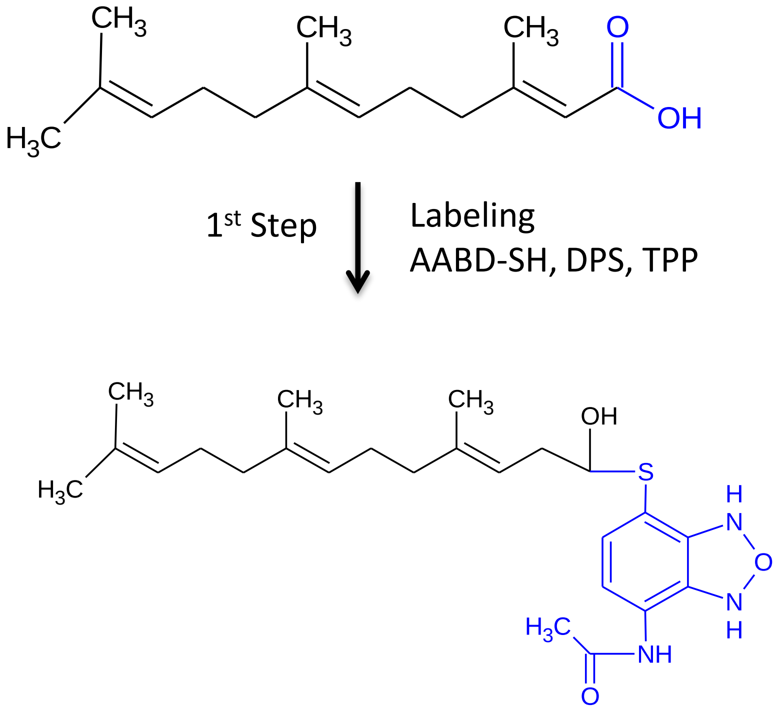

Supplement: Figure S1 — Fluorescent tagging of JH III and FA. Tagging JH III: Tagging of JH III required a two-step reaction. First step: Opening of the epoxide ring (blue) with sodium sulfide at 55°C to form a JH diol. Second step: Derivatizing with DBD-COCl to form a higher-molecular-weight fluorescent derivative (the fluorescent tag is shown in blue). Tagging FA: The carboxylic group of FA (blue) was derivatized with AABD-SH at room temperature in the presence of triphenylphosphine (TPP) and 2,2′-dipyridyl disulfide (DPDS). The process resulted in the formation of a higher-molecular-weight fluorescent derivative (the fluorescent tag is shown in blue). (PDF) [file pone.0043784.s001.pdf]
