# Supplementary figures and images for: A Quantitative Assay for the Juvenile Hormones and Their Precursors Using Fluorescent Tags
Source: PLoS One. 2012 Aug 22;7(8):e43784. doi: 10.1371/journal.pone.0043784 (PMC3425502; doi:10.1371/journal.pone.0043784)

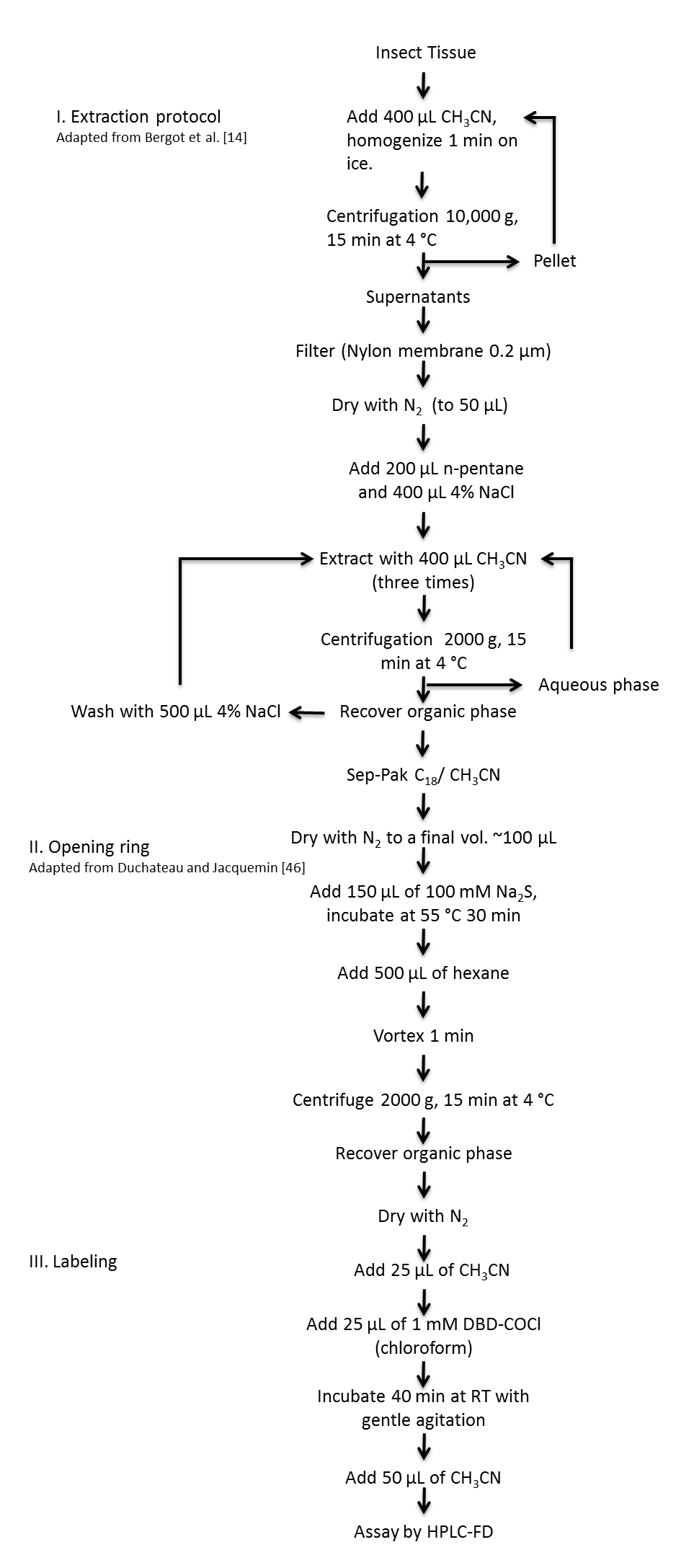

Supplement: Figure S2 — In vitro labeling JH III from biological samples. I) Extraction protocol: Insect tissues were processed by the method described by Bergot et al. (1981) that includes an acetonitrile/pentane extraction and a C18 solid-phase extraction cartridge purification. The recovered organic fraction was reduced to a volume of a 100 µl and the JH III epoxide ring was opened by the addition of 150 µl of sodium sulfide and incubation at 55°C for 30 min. Samples were then extracted with hexane; the recovered organic phase (∼500 µl) was filtered with a Nalgene filter (0.2 µm nylon membrane), dried under N2 and stored at −20°C until used. II) Opening epoxide ring: The epoxide ring was opened by the method described by Duchateau and Jacquemin (1993). After extraction, 150 µl of 100 mM sodium sulfide was added and the epoxide ring was opened by heating the biological extracts for 30 min at 55°C. Afterwards, samples were extracted using hexane. The recovered organic phase (∼500 µl) was filtered with a Nalgene filter (0.2 µm nylon membrane) and dried under N2 atmosphere. III) Labeling with a fluorescent tag: For fluorescent tagging, samples were reconstituted with 25 µl of acetonitrile and 25 µl of 1 mM DBD-COCl were added. Labeling mixtures were incubated at room temperature for 40 min and reactions were terminated by adding 50 µl of acetonitrile. Aliquot of the reactions were analyzed by HPLC-FD. (TIF) [file pone.0043784.s002.tif]

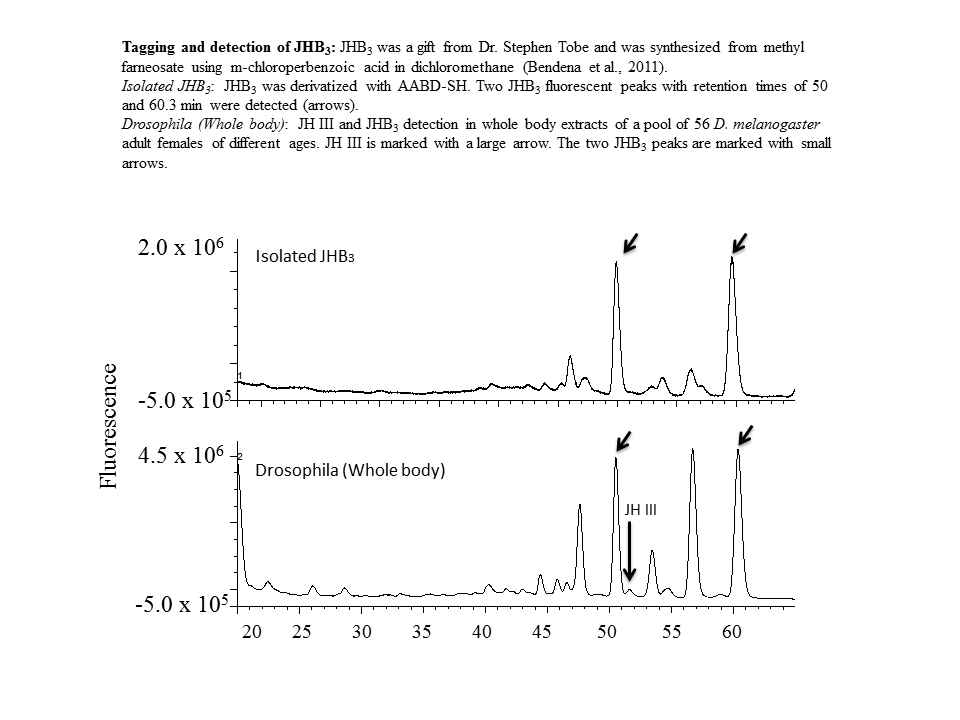

Supplement: Figure S3 — Tagging and detection of JHB3. JHB3 was a gift from Dr. Stephen Tobe and was synthesized from methyl farnesoate using m-chloroperbenzoic acid in dichloromethane (Bendena et al., 2011). Isolated JHB3: JHB3 was derivatized with AABD-SH. Two JHB3 fluorescent peaks with retention times of 50 and 60.3 were detected (arrows). Drosophila (whole body): JH III and JHB3 detection in whole body extracts of a pool of 56 D. Melanogaster adult females of different ages/JH III is marked with a large arrow. The two JHB3 peaks are marked with small arrows. (TIF) [file pone.0043784.s003.tif]

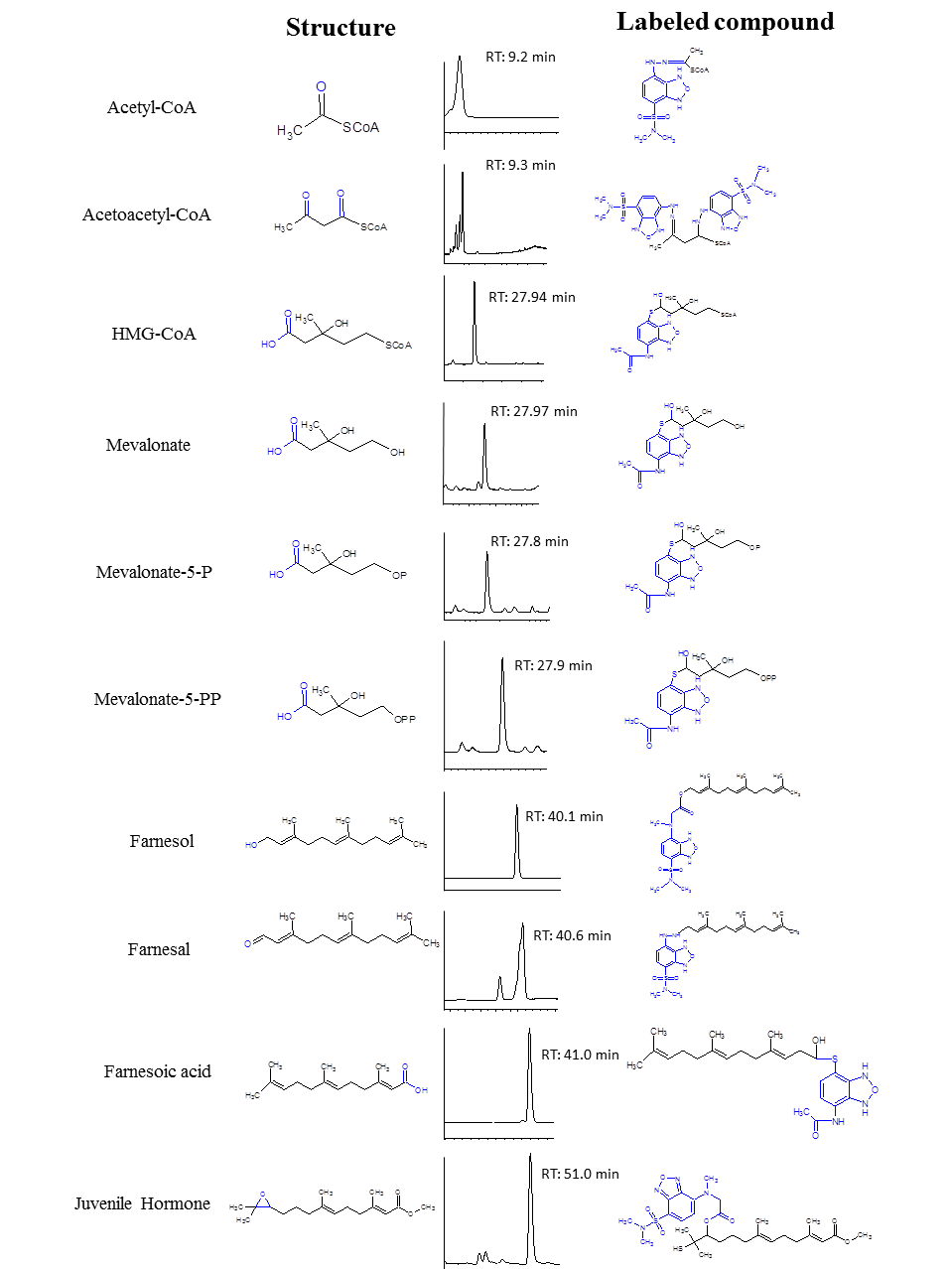

Supplement: Figure S4 — JH pathway precursors derivatized with fluorescent tags. A variety of additional functional groups can be targeted to add fluorescent tags to the other JH III precursors. We labeled and detected the thiol group of acetyl-CoA and acetoacetyl-CoA with DBD-H ( = 4-(N,N-Dimethylaminosulfonyl)-7-hydrazino-2,1,3-benzoxadiazole) at Exc/Em: 450/565 nm, the hydroxyl group of farnesol with DBD-COCl (4-(N,N-Dimethylaminosulfonyl)-7-(N-chloroformylmethyl-N-methylamino)benzofurazan) at Exc/Em: 443/546, the carboxyl group of HMG-CoA, mevalonate, phosphomevalonate and diphosphomevalonate with AABD-SH ( = 4-acetamido-7-mercapto-2,1,3-benzoxadiazole) at Exc/Em: 368/524 nm, and the aldehyde group of farnesal with NBD-H ( = 4-hydrazino-7-nitro-2,1,3-benzoxadiazole hydrazine) at Exc/Em: 450/565. The precursor were eluted by reverse phase-HPLC coupled with a fluorometer detector at the same conditions described for JH and farnesoic acid. (TIF) [file pone.0043784.s004.tif]
